# New Medical Journals

**Published:** 1853-09

**Authors:** 


NEW MEDICAL JOURNALS.

We have three new medical journals on our table, of which the following are the titles: "*The Medical Reporter*; a quarterly Journal published under the direction of the Chester and Delaware County Medical Societies;" "The *Iowa Medical Journal*; conducted by the Faculty of the Medical Department of the Iowa University;" and the *Peninsular Journal of Medicine and the Collateral Seiences*; edited by E. Andrews, A. M., M. D., Demonstrator of Anatomy in the University of Michigan."

The first of these is an emanation of medical societies and will consist of their transactions. The editors remark, that "within the bounds of the two societies, a great many interesting cases of disease are lost to the professional reader, for the want of a more convenient opportunity of publication. To record these cases—to circulate biographical notices of those deceased physicians who have acquired among us a reputable standing in the profession—to foster a more general interest in the dissemination of medical science, and to stimulate and bring into fuller action the medical talent within our limits, are the principal incentives to the publication of this Journal."

The editors of the others enter upon their task with zeal, relying upon their professional brethren for contributions. We hope they may succeed in stimulating their brethren to write, and if they do, they may esteem themselves fortunate. The great difficulty in the way of successful journalism in our country, is the want of writers in proportion to the number of our publications; and the result is that we are driven to the necessity of filling our monthly issues with extracts from one another. It must be curious to those who have access to all our medical journals to remark how often the same article reapperes. This would be well enough if the readers of one journal saw no other, but where one happens to be a reader of two or three this repetition must become irksome.

